# Regulation of eIF4E guides a unique translational program to control erythroid maturation

**DOI:** 10.1126/sciadv.add3942

**Published:** 2022-12-23

**Authors:** Craig M. Forester, Juan A. Oses-Prieto, Nancy J. Phillips, Sohit Miglani, Xiaming Pang, Gun Woo Byeon, Rachel DeMarco, Al Burlingame, Maria Barna, Davide Ruggero

**Affiliations:** ^1^ Department of Pediatrics, University of Colorado, Denver, CO 80045, USA.; ^2^ Division of Pediatric Hematology/Oncology/Bone Marrow Transplant, Children’s Hospital Colorado, University of Colorado-Anschutz Medical Campus, Aurora, CO 80045, USA.; ^3^ Department of Urology, University of California, San Francisco, San Francisco, CA, USA.; ^4^ Helen Diller Family Comprehensive Cancer Center, University of California, San Francisco (UCSF), San Francisco, CA 94158, USA.; ^5^ Department of Cellular and Molecular Pharmacology, University of California, San Francisco, San Francisco, CA 94158, USA.; ^6^ Department of Biochemistry and Biophysics, University of California, San Francisco, San Francisco, CA, USA.; ^7^ Department of Genetics, Stanford University School of Medicine, Stanford, CA 94309, USA.

## Abstract

Translation control is essential in balancing hematopoietic precursors and differentiation; however, the mechanisms underlying this program are poorly understood. We found that the activity of the major cap-binding protein eIF4E is unexpectedly regulated in a dynamic manner throughout erythropoiesis that is uncoupled from global protein synthesis rates. Moreover, eIF4E activity directs erythroid maturation, and increased eIF4E expression maintains cells in an early erythroid state associated with a translation program driving the expression of PTPN6 and Igf2bp1. A cytosine-enriched motif in the 5′ untranslated region is important for eIF4E-mediated translation specificity. Therefore, selective translation of key target genes necessary for the maintenance of early erythroid states by eIF4E highlights a unique mechanism used by hematopoietic precursors to rapidly elicit erythropoietic maturation upon need.

## Teaser

eIF4E guides a unique mechanism of specific gene expression in quiescent blood precursors to retain early, immature states.

## INTRODUCTION

Erythropoiesis is an intricately orchestrated process of hematopoiesis responsible for the production of nearly 1 × 10^11^ new mature red blood cells daily. This process is tightly regulated to balance the maintenance of erythroid progenitors with terminal differentiation to meet red blood cell demand (*1*, *2*). Physiologic challenges require the erythropoietic system to be capable of rapidly responding to cues indicative of stresses such as hypoxia and anemia (*3*). Gene expression at the translation level is uniquely suited to dictate protein dosage rapidly and robustly in response to extracellular cues (*4*). Changes in global protein synthesis rate have been reported to be important in maintaining hematopoietic stem cell (HSC) populations and in fetal erythroid maturation (*5*, *6*). However, how translational control fine-tunes proteomic networks that underlie crucial cell fate decisions from precursors cells to more differentiated erythroid cells remains an outstanding question.

Signaling in the bone marrow through the cytokines stem cell factor (SCF) and Erythropoietin (Epo) cue gene expression through multiple pathways including phosphatidylinositol 3-kinase (PI3K), Akt, and Stat5 (*1*, *7*). Downstream of the aforementioned signaling pathways lies the master regulator of protein synthesis, mammalian target of rapamycin (mTOR). mTORC1, the complex of mTOR with its binding partner Raptor, has come to the forefront of HSC fates including regulation of proliferation, stem cell maintenance, and autophagy programs (*5*, *8*–*10*). Epo- and SCF-induced signaling through mTOR leads to phosphorylation of important regulators of translation such as p70S6 kinase and 4EBP1, the inhibitor of eIF4E (initiation factor 4E) activity (*11*). Specifically, phosphorylation of 4EBP1 leads to release and activation of eIF4E, the major cap-binding protein, thus allowing eIF4E to interact with other translation factors to recruit the ribosome to the 5′ untranslated region (5′UTR) of mRNA. eIF4E is a key node for translational control and is a hub integrating multiple signaling pathways including PI3K-mTOR and Ras/mitogen-activated protein kinase (*12*). Activity of eIF4E in erythropoiesis has been implicated in the regulation of multiple events such as translation of *GATA1*, *Igbp1*, *LAT3*, and *RBM38* (*13*–*16*). To uncover how specific mRNAs are targeted for translation, we focused on eIF4E. Once held to be the rate-limiting step in global translation via recruitment of the ribosome, eIF4E directs the synthesis of specific proteins in a cellular context–dependent manner, thus creating an ideal target to study in early erythropoiesis (*17*–*19*).

In this study, we characterized a dynamic progressive repression of eIF4E activity by hypophosphorylation of 4EBP1 throughout erythropoiesis. However, this fluctuation is not correlated with global protein synthesis activity in vivo. Failure to repress eIF4E activity through constitutive expression of eIF4E in a model of human erythropoiesis delays exit from early precursor states. This leads to aberrant erythroid progression that promotes a specific proteomic program responsible for maintaining early erythroid markers. Gene expression enriched by increased eIF4E is correlated with a cytosine-enriched motif located in the 5′UTR of target genes, which is necessary for preferential expression of the eIF4E gene network. Overall, our data highlight a key role for eIF4E dosage in regulating hematopoietic differentiation independent of global protein synthesis and provide insight into how specific gene networks are orchestrated in cells through a translational program.

## RESULTS AND DISCUSSION

### eIF4E is dynamically controlled through erythropoiesis and is uncoupled from global protein synthesis in vivo

**Fig. 1.**
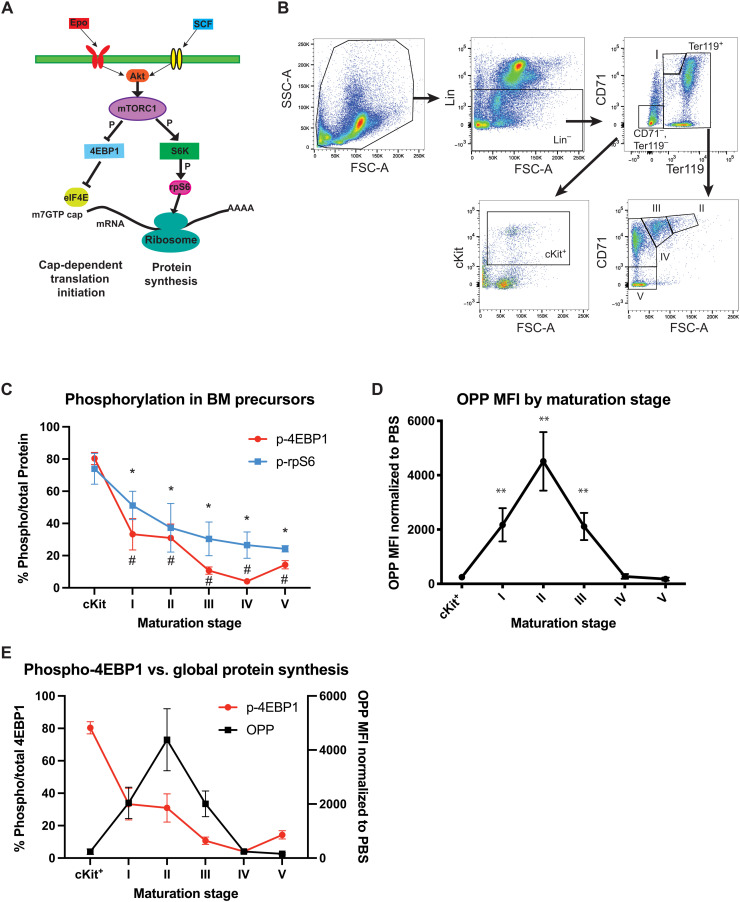
mTOR activation is dynamically down-regulated in erythropoiesis irrespective of global protein synthesis. (**A**) Downstream effectors of activated mTORC1 leading to phosphorylation of rpS6 and 4EBP1, thus derepressing eIF4E. (**B**) Flow cytometry schematic for the separation of erythroid precursors into ckit^+^ (CD71^−^, Ter119^−^, and cKit^+^), I (CD71^hi^ and Ter119^int^), II (CD71^hi^ and FSC^hi^), III (CD71^hi^ and FSC^int^), IV (CD71^hi^ and FSC^lo^), and V (CD71^lo^ and FSC^lo^). (**C**) Analysis of 8- to 12-week WT C57BL/6J mice bone marrow (BM) by phospho-flow of 4EBP1^Thr37/46^ (p-4EBP1) and rpS6^S235/236^ (p-rpS6) by the percent of positive of events expressing total 4EBP1 or rpS6 in bone marrow–derived erythroid precursors. *P < 0.05 when comparing percent of phospho-positive to total protein events of 4EBP1 or rpS6 to phosphorylation at the “ckit^+^” phase. *n* = 4. (**D**) Mean fluorescence intensity (MFI) of intraperitoneally injected OPP (1 hour) analyzed at phases of erythroid maturation. **P < 0.005 when comparing respective erythroid maturation stage to ckit^+^ precursors. *N* = 4. (**E**) Simultaneous comparison of 4EBP1 phosphorylation to global protein synthesis activity. PBS, phosphate-buffered saline.

Signaling in the bone marrow through the cytokines SCF and Epo fine-tunes gene expression through multiple pathways funneling to downstream activation of mTOR (Fig. 1A). Analysis of available proteomic (*20*) (fig. S1A) and transcriptomic (*21*) (fig. S1B) datasets demonstrates a progressive decrease (both in the protein and mRNA levels of mTOR effector arms including eIF4E, 4EBP1, and rpS6) in comparison to that of canonical up-regulation of hemoglobin subunits during erythropoiesis. We turned our focus to how posttranslational signaling events alter the activity of key regulators of translation such as rpS6 and 4EBP1 throughout erythropoiesis. Specifically, phosphorylation of 4EBP1 leads to the release and activation of eIF4E, thus allowing eIF4E to interact with other translation factors to recruit the ribosome to the 5′UTR of mRNA (Fig. 1A). To investigate whether 4EBP1 phosphorylation exerts repression of eIF4E in specific stages of erythroid maturation in vivo, we used a phospho-flow approach to analyzing arms of mTOR activation. Using a combination of the markers CD71 and Ter119 in Lineage-depleted, nucleated murine bone marrow to parse erythroid maturation into six stages (*22*) (Fig. 1B), we analyzed the activation of the signaling arms of mTOR (rpS6 or 4EBP1) by phospho-flow. Our phospho-flow approach detects sensitive alterations in phosphorylation signal of phospho-4EB1 and phospho-rpS6 that could be ablated in HPC-7 hematopoietic cells (fig. S1C) and confirmed by Western blotting (fig. S1D) by the selective and potent mTOR inhibitor pp242 (*23*). Notably, we identified dynamic changes in phosphorylation of 4EBP1 and therefore eIF4E activity throughout erythropoiesis (Fig. 1C and fig. S1E). Hypophosphorylation of 4EBP1 occurs along with the rpS6 arm of mTOR signaling, and both are progressively less phosphorylated with terminal erythroid maturation (Fig. 1C and fig. S2, B and C). In addition, activation of mTOR measured by phosphorylation phenocopied the pattern seen in its downstream arms 4EBP1 and rpS6 (fig. S2A).

To ask whether this dynamic hypophosphorylation led to changes in total protein synthesis, we used *O*-propargyl puromycin (OPP), a nontoxic, quantitative tool to analyze relative rates of protein synthesis in vivo (*24*). The 4EBP1-mediated repression was not accompanied by a concurrent decline in overall global translation (Fig. 1D). These data imply that eIF4E is more available to direct translation in early, noncommitted erythroid precursors (cKit^+^) with low global protein synthesis but becomes negatively regulated upon erythrocyte maturation (III) (Fig. 1E). The balance of eIF4E activity potentially gives a unique insight into how the translatome is remodeled between immature cell types and during their differentiation.

### Increased eIF4E expression impairs early erythroid maturation

**Fig. 2.**
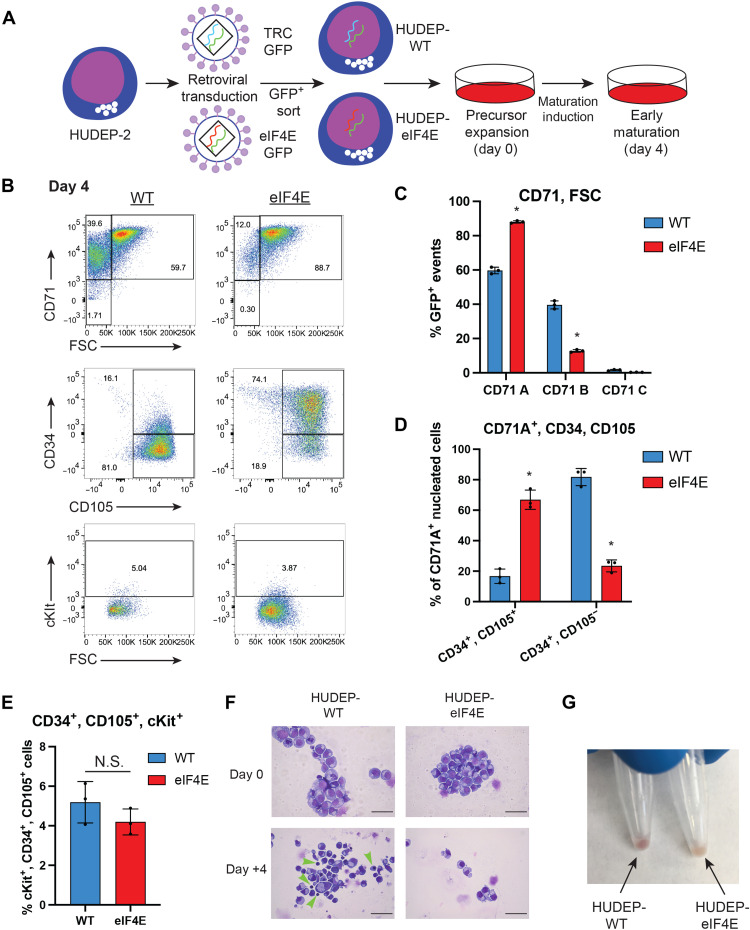
Constitutive expression of eIF4E impairs erythroid maturation in HUDEP-2 cells. (**A**) HUDEP-2 cells transduced with retrovirus encoding eIF4E [pMSCV-eIF4E-IRES-GFP (green fluorescent protein)] or The RNAi Consortium (TRC) control (pMSCV-TRC-IRES-GFP) followed by GFP sorting, precursor expansion, and differentiation. (**B**) Representative flow cytometry plots comparing HUDEP-WT and HUDEP-eIF4E cells 4 days after induction of maturation. (**C** to **E**) Quantitation of flow cytometry–defined differences in maturation between WT and eIF4E HUDEP-2 cells by (C) CD71 and FSC, (D) CD34 and CD105, and (E) cKit. *P < 0.05, *N* = 3. (**F**) Representative Wright-Giemsa–stained cytospins of day 0 and +4 HUDEP-WT and HUDEP-eIF4E cells displaying relative homogeneity in day 0 of both conditions (top) and day +4 (bottom) showing marked anisocytosis in HUDEP-WT cells compared to HUDEP-eIF4E cells. Green arrowheads denote erythroblasts showing nuclear condensation and size restriction. Magnification, ×40. Black line indicates size marker of 100 μm. (**G**) Representative day +4 cell pellet of WT versus eIF4E HUDEP-2 cells showing impaired hemoglobinization evidenced by the pale color of the pellet. Statistical analysis between populations using paired *t* test compared to HUDEP-WT. N.S., not significant.

Taking into consideration that eIF4E activity is uncoupled from changes in global protein synthesis, we hypothesized that eIF4E activity directs a translational program driving specific targets associated with early erythroid states that must be repressed to facilitate differentiation. To test our hypothesis, we used HUDEP-2 cells, a human cord blood–derived, nontransformed cell line capable of expanding as early precursors and differentiating into mature erythroid cells (*25*). In addition, HUDEP-2 cells display a similar pattern of 4EBP1 progressive hypophosphorylation upon cues to differentiate toward mature erythroid cells (fig. S2D). To evaluate the functional relevance of decreased eIF4E activity in erythropoiesis, we retrovirally transduced HUDEP-2 cells with either an empty vector [HUDEP-WT (wild type)] or eIF4E overexpression (HUDEP-eIF4E) and induced them to differentiate (Fig. 2A). Enhanced expression of eIF4E during expansion did not significantly alter markers of early erythroid precursor states constituting a CD71^+^ population of homogeneous size, with no significant differences in early markers including CD34, CD105, and cKit (fig. S2, E to I) (*26*, *27*). However, at day +4 after differentiation (24 hours after the removal of SCF from culture media), eIF4E-overexpressing cells were impaired in differentiation, retaining early precursor markers characterized by the retention of a CD71^+^, FSC^hi^ population along with increased expression of CD34^+^ and CD105^+^ compared to that of HUDEP-WT cells (Fig. 2, B to E). These differing markers correspond to an alteration in cell morphology toward a homogeneous precursor in HUDEP-eIF4E compared to the heterogeneous array of maturing erythrocytes in the HUDEP-WT cells represented by an increase in smaller (FSC^lo^, CD71^+^) cells denoted as population “B” (Fig. 2, B, C, and F). In addition, overexpression of eIF4E delays hemoglobinization of maturing HUDEP-2 cells at day +4 after differentiation induction (Fig. 2G). Impairment in early erythroid maturation was supported by retrovirally mediated eIF4E overexpression in primary human CD34^+^ cells, leading to accumulation in CD235^+^, CD49d^hi^, CD233^lo^ precursors (*28*) at day 6 after erythroid maturation induction (fig. S3, A to C). Collectively, these findings implicate high eIF4E activity in maintaining markers of early erythroid maturation despite erythroid-directed maturation cues.

### eIF4E directs the expression of an early erythroid gene network

**Fig. 3.**
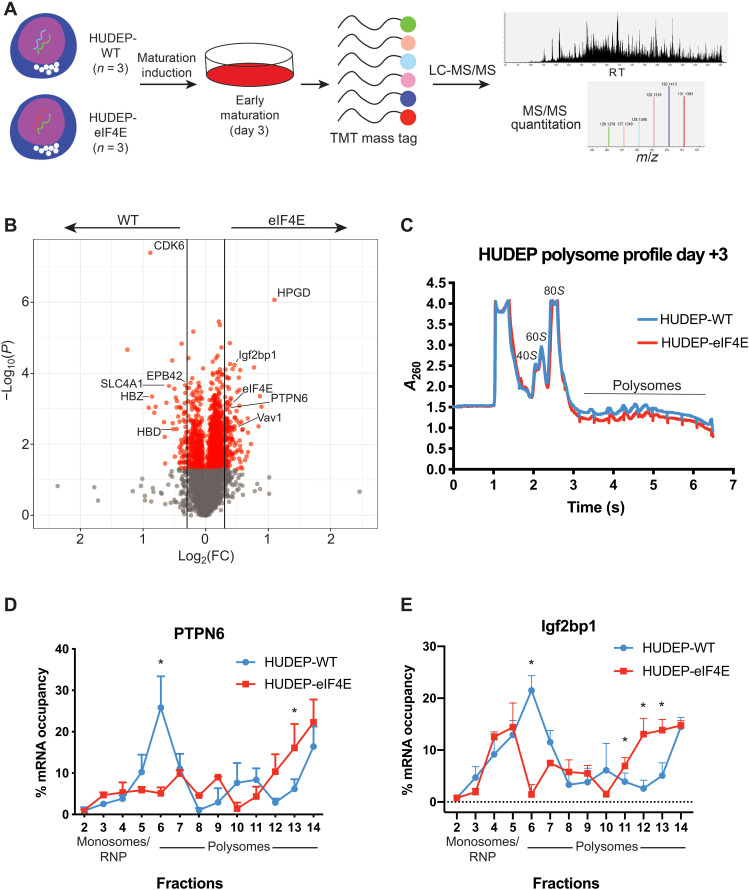
eIF4E constitutive expression rewires proteomic program in differentiating HUDEP-2 cells. (**A**) HUDEP-WT and HUDEP-eIF4E cells induced to mature and harvested in triplicate at day +3 and then subjected to TMT isobaric mass tag–labeled quantitative MS. Representative total ion chromatogram showing MS peak intensity versus retention time (RT) and an expansion of the TMT reporter ion mass/charge ratio (*m*/*z*) region of a peptide tandem MS (MS/MS) spectrum. LC, liquid chromatography. (**B**) Volcano plot displaying relative protein levels in HUDEP-eIF4E versus HUDEP-WT cells as log_2_ FC against −log_10_(*P* value). Proteins with significantly altered levels between the two states (*P* < 0.05) are labeled in red (*P* > 0.05, gray). Cutoff for significant log_2_(FC) > 0.3. (**C**) Comparison of polysome trace profiles of HUDEP-WT versus eIF4E displaying relative similarity of ribosomal biogenesis between cellular types. Polysome/monosome ratio of HUDEP-WT = 0.8383 ± 0.06 and 0.6626 ± 0.17 in HUDEP-eIF4E cells. *A*_260_, absorbance at 260 nm. (**D**) Quantitative polymerase chain reaction (qPCR) of *Ptpn6* at specified polysome fractions in HUDEP-WT versus HUDEP-eIF4E. Asterisk indicates adjusted *P* < 0.05 by Bonferroni-Sidak method with α = 0.05. (**E**) qPCR of *Igf2bp1* at specified polysome fractions in HUDEP-WT versus HUDEP-eIF4E. Asterisk indicates adjusted *P* < 0.05 by Bonferroni-Sidak method with α = 0.05.

To identify the gene expression program orchestrated by eIF4E allowing the preservation of early erythroid phenotypic markers even after SCF withdrawal in HUDEP cells, we performed proteome-wide Tandem Mass Tag (TMT) isobaric mass tag labeling of proteins at day +3 to quantitatively compare the proteins up-regulated by eIF4E in HUDEP-2 cells by mass spectrometry (MS; Fig. 3A). Although HUDEP-eIF4E cells do not display marked differences in immunophenotypic markers at day +3 (fig. S3D), we identified a total of 6303 unique proteins of which 143 proteins were increased in HUDEP-4E versus 43 increased in HUDEP-WT cells [log_2_FC (fold change) > 0.3, *P* < 0.05; tables S1 and S4]. Quantitation by MS demonstrated an increase of 0.3395 log_2_ FC of eIF4E expression in HUDEP-4E compared to HUDEP-WT (table S1). Analysis of eIF4E-directed genes shows the up-regulation of targets implicated in early hematopoiesis such as PTPN6 (*29*), IGF2BP1 (*30*), VAV1 (*31*), and SOX6 (*32*), whereas multiple genes crucial in maturation (*26*) including HBD, HBB, HBZ, CD44, GYPA, and SLC4A1 were enriched in HUDEP-WT cells (Fig. 3B). While analysis of gene ontology (GO) programs up-regulated in HUDEP-WT cells corresponded to protein synthesis and hemoglobinization inherent to maturing erythrocytes, these classes were not enriched in HUDEP-4E cells, which were instead enriched in functions responsible for intracellular trafficking and phospholipid binding (fig. S3E).

However, how eIF4E mechanistically exerts this change of gene expression was unclear. Protein abundance is a culmination of transcription rates, translation efficiency, and rates of degradation (*33*). Reevaluation of available transcriptomic (*21*) (fig. S3F) and proteomic datasets (fig. S3G) (*20*) demonstrates a progressive decline in identified eIF4E targets across human erythroid maturation. Of particular interest among eIF4E targets, *PTPN6* is a tyrosine phosphatase known to regulate early erythropoietic progression (*29*), and *IGF2BP1* is an RNA binding protein with described roles in stem cells and early erythroid gene expression (*30*, *34*). Quantitation of transcripts in WT HUDEP maturation shows a decline in PTPN6 with a relatively stable Igf2bp1 transcript abundance (fig. S4A), while protein levels appear to decline slightly for both (fig. S4B). However, comparison of mRNA transcript level of an array of genes up-regulated by eIF4E including *PTPN6*, *VAV1*, *IGF1BP1*, *FTH*, *FTL*, and *CD63* did not demonstrate up-regulation in the HUDEP-eIF4E population. (fig. S4C). Increased eIF4E did not alter the posttranslational stability of up-regulated targets as neither PTPN6 nor IGF2BP1 displayed significant enhanced accumulation after cycloheximide pulse treatment in HUDEP-4E cells (fig. S4, D and E). Despite minimal changes in immunophenotypic markers at day 0 before induction of maturation (fig. S2, F to I), HUDEP-eIF4E cells express increased PTPN6 and Igf2bp1 (fig. S4F), suggesting that eIF4E “primes” precursors to retain early, immature fates. To dissect mechanisms underlying specificity in eIF4E-directed gene expression in erythroid maturation, we used polysome profiling by sucrose gradient to evaluate the effects on transcript-specific translational control. The overall polysome profiles were the same between HUDEP-WT and HUDEP-4E conditions, revealing that global changes in translation are not affected by eIF4E expression (Fig. 3C). Supporting this notion, increased eIF4E expression did not correlate with enhanced OPP incorporation at day +3 after induction of maturation (fig. S3G). However, we found that PTPN6 and IGF2BP1 mRNAs were bound to actively translating, heavy polysomes in HUDEP-4E cells (Fig. 3, D and E), while they remained predominantly bound to less translationally active, light polysomes in HUDEP-WT cells. β-Actin, a protein whose expression was not favored by either WT or eIF4E conditions, did not show a similar change in sucrose gradient fractionation (fig. S3H).

### eIF4E regulates early erythroid maturation through downstream up-regulation of Igf2bp1 and PTPN6

**Fig. 4.**
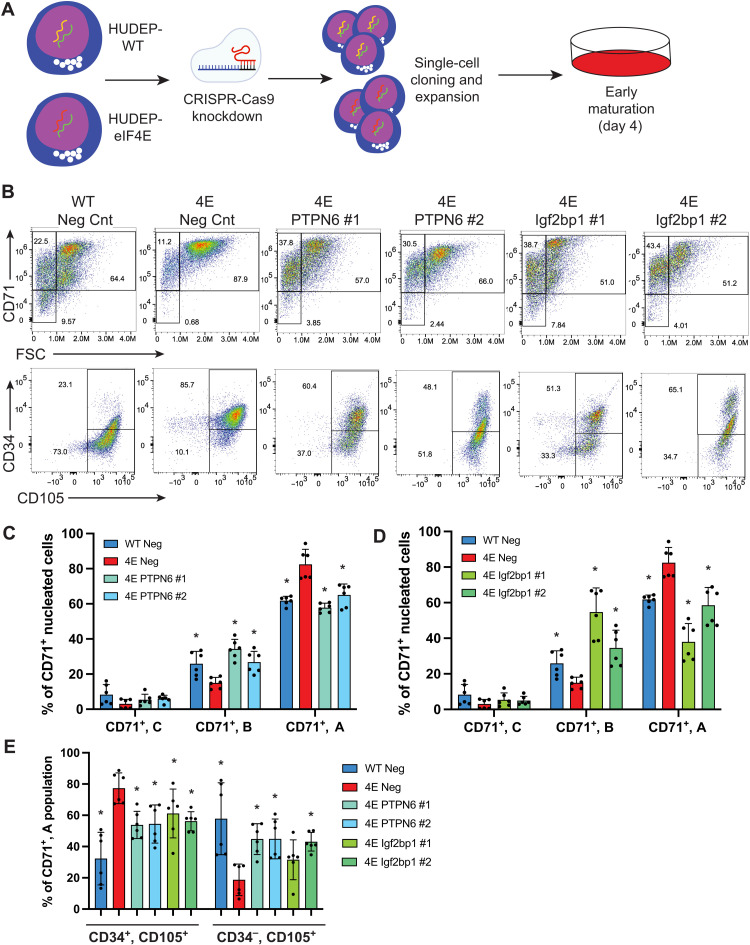
Delay in eIF4E-mediated erythroid maturation dependent on PTPN6 and Igf2bp1. (**A**) HUDEP-2 WT and eIF4E cells nucleofected with RNP containing Cas9 with crRNA:tracrRNA duplex specific to either noncoding negative control, PTPN6, or Igf2bp1. Single-cell clones were generated and expanded before induction of maturation. (**B**) Representative flow cytometry plots of CD71 versus FSC and CD34 versus CD105 expression demonstrating the reversal of erythroid maturation in HUDEP-eIF4E (negative CRISPR control) cells with simultaneous knockdown of PTPN6 (4E PTPN6 1 or 4E PTPN6 2) or Igf2bp1 (4E Igf2bp1 1 or Igf2bp1 2) in comparison to HUDEP-WT (negative CRISPR control). (**C**) Partial rescue of CD71^+^ erythroid maturation phases dependent on PTPN6 knockdown. *P < 0.05, *N* = 6. (**D**) Partial rescue of CD71^+^ erythroid maturation phases dependent on Igf2bp1 knockdown. *P < 0.05, *N* = 6. (**E**) Partial rescue of CD34^+^, CD105^+^ and CD34^−^, CD105^+^ populations with knockdown of PTPN6 or Igf2bp1. *P < 0.05, *N* = 6. Statistical analysis between populations calculated using paired *t* test compared to HUDEP-4E negative control (4E Ng Cnt).

To investigate whether proteins up-regulated by eIF4E are sufficient to confer the phenotype of impaired erythroid maturation, we focused on two proteins increased in HUDEP-4E cells and implicated in the regulation of early hematopoiesis, PTPN6 and IGF2BP1. Using CRISPR-Cas9 gene editing (*35*), HUDEP-WT or HUDEP-4E cells were nucleofected with a ribonucleoprotein (RNP) containing either a negative control CRISPR-RNA (crRNA) designed to not have homology to genomic targets in human (Neg) or two unique crRNAs per gene targeting either PTPN6 or Igf2bp1. Single-cell clones were isolated and expanded before knockdown confirmation by immunoblotting and maturation (Fig. 4A and fig. S5A). Single-cell CRISPR knockdown clones demonstrated robust efficiency in targeted reduction of protein abundance in selected clones with minimal expression of the targeted gene by either crRNA against both PTPN6 and Igf2bp1 (fig. S5A). Maturation of HUDEP-4E cells harboring knockdown of PTPN6 or IGF2BP1 displayed a shift toward cell size heterogeneity seen in HUDEP-WT cells (Fig. 4, B to D) and partial rescue of reduced CD34 expression (Fig. 4E). Quantitative polymerase chain reaction (qPCR) of canonical mature erythroid gene targets in knockdown rescue experiments of HUDEP-eIF4E cells demonstrated restoration of WT levels of β-globin, spectrin A, ankyrin, and band 4.2 by Igf2bp1 knockdown with rescue of β-globin and ankyrin by PTPN6 knockdown (fig. S5B). These targeted rescue studies are supportive of the dependence of PTPN6 and Igf2bp1 on promoting erythroid maturation.

### mRNA up-regulated by eIF4E harbors a conserved 5′UTR cytosine-rich motif conferring enhanced translation

**Fig. 5.**
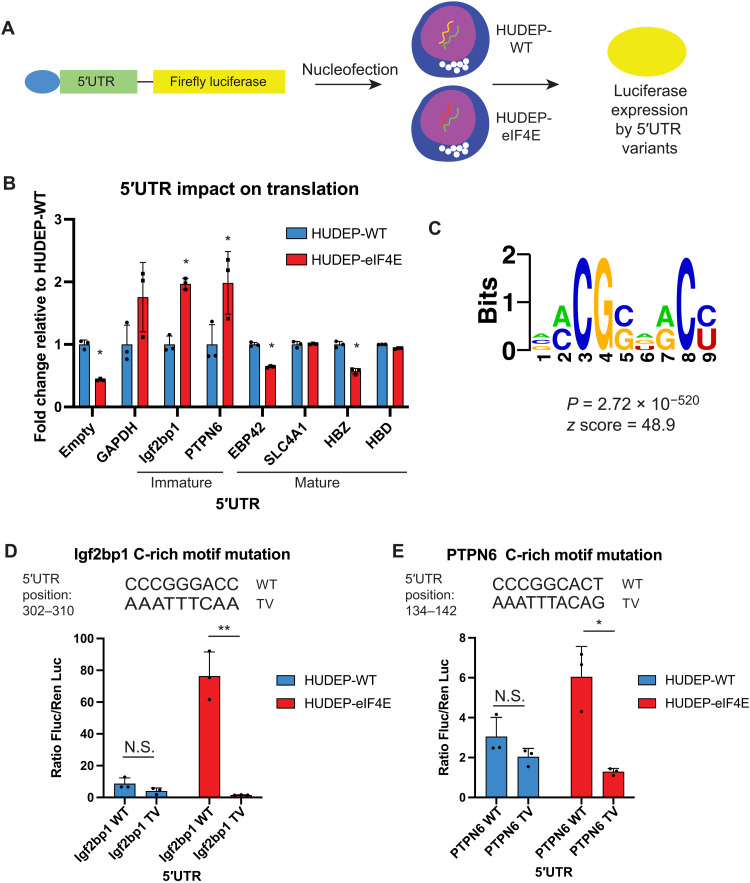
Dependence of conserved C-rich motif in 5′UTR of genes up-regulated by eIF4E. (**A**) Firefly luciferase reporter constructs cloned with 5′UTR of identified genes were nucleofected into HUDEP-WT or HUDEP-eIF4E cells, and luciferase was analyzed as a readout of translation efficiency from the respective 5′UTR. (**B**) Comparison of luciferase expression as FC relative to HUDEP-WT in each respective luciferase reporter construct. “Immature” denotes genes associated with early erythroid precursor states. “Mature” denotes genes associated with maturing erythrocytes. *P < 0.05, *N* = 3. GAPDH, glyceraldehyde phosphate dehydrogenase. (**C**) Comparison of eIF4E–up-regulated genes against a set of shuffled sequences of the respective of 5′UTR identifies a conserved 9-nt C-rich motif in 90 of 143 genes. (**D**) Comparison of luciferase expression driven by Igf2bp1 5′UTR as a ratio of Firefly/*Renilla* luciferase comparing WT 5′UTR (WT) versus 5′UTR harboring a nucleotide transversion of a 20-nt segment encompassing the C-rich motif (TV) after nucleofection into HUDEP-WT or HUDEP-eIF4E cells. **P < 0.005. *N* = 3. “WT” denotes WT 5′UTR sequence. “TV” denotes nucleotide transversion sequence mutation. (**E**) Similar strategy to that in (D) except the comparison of PTPN6 5′UTR. *P < 0.05. *N* = 3. Statistical analysis between populations calculated using paired *t* test.

To further delineate how eIF4E recognizes transcripts for translation in specific erythroid maturation phases, we delved deeper into sequence determinants. Motifs contained within the 5′UTR of mRNAs confer a significant role in translational efficiency (*36*), some of which directly rely on eIF4E activity (*37*). To determine whether elements contained in the 5′UTR of candidate genes were sufficient to dictate specificity in translation efficiency, 5′UTR sequences were cloned upstream of Firefly luciferase (pGL3-SV40) and conucleofected with *Renilla* luciferase (pRL-SV40) into HUDEP-WT and HUDEP-4E cells on day +3 after differentiation and analyzed 16 hours afterward (Fig. 5A). HUDEP-4E cells displayed enhanced luciferase expression directed by the 5′UTR of Igf2bp1 or PTPN6, while HUDEP-WT cells promoted increased luciferase expression directed by the 5′UTR from targets including EBP42 (*38*) and HBZ (*39*), both of which are associated with maturing erythrocytes (Fig. 5B). While compelling that elements in the specific 5′UTRs were sufficient to promote translation depending on eIF4E expression levels, we globally examined whether selective properties of 5′UTRs suggested patterns in eIF4E recognition. Comparison of calculated biochemical properties of 5′UTRs from genes up-regulated in WT or eIF4E backgrounds displayed a trend toward higher GC content, minimum free energy (MFE), and lower sequence length in 5′UTR up-regulated by eIF4E (fig. S6, A to C). However, normalization of MFE to respective 5′UTR length negated differences in MFE (fig. S6D), and therefore, we evaluated whether unique sequence elements guide translation specificity by eIF4E. To determine whether eIF4E translation targets may be regulated by sequence elements in their 5′UTR, we used the sequence motif discovery algorithm FIRE (*40*) to detect conserved motifs used by eIF4E in target mRNA 5′UTRs. We performed analysis of the 5′UTR genes up-regulated by WT and eIF4E genotype backgrounds (table S4) for differential enrichment of sequence motifs. This interrogation yielded a cytosine-rich, 9-nucleotide (nt) motif in 90 of 143 genes up-regulated by eIF4E (Fig. 5C). A similar approach analyzing the 5′UTRs from transcripts corresponding to the proteins up-regulated in HUDEP-WT did not yield a statistically significant motif (fig. S6E). Dependence of this motif on translation control directed by eIF4E was analyzed by comparison of WT 5′UTRs versus mutant 5′UTRs harboring nucleotide transversion of the C-rich motif of candidate transcripts fused to a luciferase reporter. Evaluation of luciferase expression by 5′UTR variants showed that up-regulated translation by increased eIF4E is dependent on the C-rich motif as nucleotide transversion of the C-rich motif markedly reduced the expression of both the PTPN6 and Igf2bp1 5′UTRs in HUDEP-4E cells (Fig. 5, D and E). These data suggest that eIF4E dosage is important to control the translation of specific gene networks through nucleotide-specific motifs located in the 5′UTRs of target mRNAs. Therefore, eIF4E levels must be tightly controlled to prevent the untimely translation of these target mRNAs during erythroid differentiation.

In this study, we highlight a role for eIF4E activation in steering the translation of unique gene expression networks as a mechanism to specify early erythroid maturation. Our work expands the concept of eIF4E-mediated gene expression occurring through targeting of specific transcripts independent of global protein synthesis rates in a model of hematopoietic differentiation. Early, immature phases of erythroid maturation are composed of a relatively similar diversity of known transcribed genes and prior to marked surges in differential gene expression occurring in the late basophilic erythroblast phase (*21*, *41*). We find that eIF4E activity guides the specific translation of this repertoire to maintain immature erythroid states where the nucleus is still intact. In addition, selective advantage in translation efficiency conferred by eIF4E activity can be localized to a conserved cytosine-rich motif displaying exquisite sensitivity to nucleotide composition. Concise 5′UTR sequence motifs including the pyrimidine-rich translational element (PRTE) (*37*), 5′-terminal oligopyrimidine (5′TOP) (*42*), or cytosine-enriched translational element (CERT) (*17*) drive the translation of specific transcripts, all of which are known translational regulatory elements controlled by eIF4E. Our motif differs from the 5′TOP and PRTE motifs in that it lacks pyrimidine enrichment and does not harbor the location specificity of 5′TOP (average position of our motif is 197 nt downstream of 5′ cap). This motif most closely resembles the CERT motif by the lack of position dependence and GC-rich composition. Whether the 9-nt motif identified in our studies represents a CERT-like motif and whether eIF4E interacts directly with this motif remain to be established. This motif may be a docking site for an unknown cofactor of eIF4E, although prediction of verified binding motifs of a cohort of RNA binding proteins using RBPsuite (*43*) does not overlap with the identified motif in PTPN6 or Igf2bp1. In silico prediction of RNA secondary structure localized within the conserved motif predicted potential stem-loop structures (fig. S6, F and G). However, further research into the role of this motif, potential RNA binding partners, and the contribution of RNA secondary structure may deepen our understanding of the mechanisms of translational control in differentiation and lead to the discovery of novel targets to facilitate hematopoietic expansion.

We find a prolonged expression of early erythroid markers even after withdrawal of SCF from maturation conditions (Fig. 2, D and E) upon eIF4E overexpression. Here, we further define the molecular determinants of the 5′UTR inherent to translational specificity important for the balance of early erythroid precursors and differentiated erythroid cells. Activation of eIF4E has a well-characterized role in cell proliferation and antiapoptosis and is considered a proto-oncogene (*44*). However, in erythropoietic maturation, we observe a preservation of early immature states by eIF4E activity even after withdrawal of HSC stemness-promoting SCF (*45*), supporting a role for eIF4E in differentiation. As SCF-dependent signaling is sufficient to confer complete phosphorylation of 4EBP1 (*46*), increased eIF4E activity may overcome lack of SCF signals without increases in global protein synthesis. However, the role of eIF4E-dependent translation in differentiation is still poorly understood. Prior work has dissected how eIF4E activity discriminates selective translation of key isoforms of genes involved in differentiation including GATA1 (*13*) and YY2 (*47*). In addition, studies have implicated eIF4E in controlling the differentiation of cerebellar granule neuron precursors (*48*). However, what specific mRNA networks eIF4E controls in this context remain unknown. The culmination of these and our work represents an emerging field to understand how eIF4E engenders progrowth pathways in specific cellular contexts and retention of immaturity in others by orchestrating unique translation programs.

We identified specific up-regulation of a network of proteins by eIF4E and focused further on PTPN6 and Igf2bp1 to explore deeper into how eIF4E maintains early precursor states. The protein tyrosine phosphatase PTPN6 is primarily expressed in hematopoietic development and exerts inhibitory effects on tyrosine kinases by direct dephosphorylation of target substrates involved in growth pathways. More specifically, PTPN6 counteracts erythroid progression driven by Epo (*49*) and maintains HSC quiescence through transforming growth factor–β signaling (*50*). Igf2bp1 is an mRNA binding protein regulating an array of mechanisms controlling gene expression primarily through the recognition of RNA m^6^A epitranscriptomic modifications. Igf2bp1 plays a key role in establishing fetal erythroid programs through up-regulation of γ-globin (*51*). In addition, m^6^A methyltransferases and cognate mRNA marks play a key role in the expression of genes required for terminal erythroid differentiation (*52*). It remains unanswered whether the balance of Igf2bp1 directs early precursor states in a manner dependent on these modifications. Undoubtedly, eIF4E directs the specific translation of a wide array of mRNA contributing to the early precursor state, but the actions of PTPN6 and Igf2bp1 lend a window into the key processes that eIF4E uses.

It remains unclear and a fundamental avenue of future research in how gene expression is orchestrated following progressive dephosphorylation of 4EBP1 during erythropoiesis. One possibility is that progression to terminal, committed erythroid maturation relies upon cap-independent translation using internal ribosome entry sites (IRESs) in a subset of key mRNAs, as has been described in hypoxia (*53*), cellular differentiation (*54*), or even cancer (*55*). IRES-dependent translation is also facilitated by binding of DAP5 to these sequences to promote the translation of apoptosis-mediating mRNA (*56*). In addition to eIF4E, the eIF3 subunit complex also promotes translation initiation through guiding the interactions of ribosomes to target mRNA. Different subunits of the eIF3 complex have capabilities to bind to 5′UTR mRNA cap (*57*), stem-loop structures (*58*), or epitranscriptomic modifications (*59*) to promote translation. Alternatively, expression of trans-acting translation factors in early phases of differentiation may be dependent on high eIF4E activity to promote the translation of subsets of genes that guide immature states.

In addition, how global synthesis rates increase even as eIF4E activity declines is an important question. We did not observe changes in global protein synthesis with increased eIF4E, but similarly, heterozygous knockout mouse models of eIF4E do not display differing global translation rates (*17*) and point to eIF4E’s role in orchestrating transcript-specific selectivity in translation control. Future work on which mechanisms guide translational control to transition from immature to terminally differentiating erythrocytes will shed light on this process and reflect an exciting new line of research to understand how this key process controls cell fate.

## MATERIALS AND METHODS

### Murine bone marrow phospho-flow

WT C57BL/6J mice in equal representation of male and female at ages between 8 and 12 weeks of life were euthanized by carbon dioxide inhalation in accordance with the Institutional Animal Care and Use Committee (IACUC) protocol (#983) approval and regulations per the University of Colorado-Anschutz Medical Center. Hematopoietic cells were harvested by flushing isolated bones with cold Iscove’s modified Dulbecco medium (IMDM) and 1% bovine serum albumin (BSA), filtered over 40-μm sterile filters, washed twice with IMDM and 1% BSA, and followed immediately by fixation with 2% paraformaldehyde. Fixed marrow cells were divided into independent pools for phospho-flow analysis of 4EBP1, rpS6, or mTOR and washed twice. Cells were then stained with CD71 and Ter119 before permeabilization with ice-cold 95% methanol on ice. Samples were then washed and rehydrated before staining with surface antibodies initially with 4EBP1-biotin and rpS6-biotin antibodies and then staining with the remainder of surface markers and phycoerythrin-Cy7–conjugated streptavidin. Samples were stained for 1 hour at room temperature in the dark before analysis on a BD LSR II (Becton-Dickinson; San Jose, CA). Inhibitor studies to demonstrate the sensitivity of the phospho-flow antibodies were performed by adding a dose range of PP242 (Selleckchem) to HPC-7 cells for 1 hour before fixation and staining. Flow cytometry data were analyzed using FlowJo (BD; Ashland, OR).

### OPP detection of global protein synthesis

WT 8- to 12-week C57BL/6J mice of equal male and female representation were injected intraperitoneally with OPP [50 mg/kg (pH 6.4 to 6.6); MedChem; Monmouth Junction, NJ], and murine bone marrow was isolated 1 hour after injection as listed above. Following filtration and washes, marrow cells were stained with surface lineage markers for Lin, Ter119, CD71, and cKit. After staining, cells were washed twice and fixed with 1% paraformaldehyde on ice and then washed and permeabilized using BD Cytofix/Cytoperm Solution (Becton-Dickinson, San Jose, CA) for 20 min at room temperature. OPP-labeled polypeptides were labeled using Click-IT cycloaddition for 30 min at room temperature using Alexa Fluor 555–azide (Click-IT Plus Alexa Fluor Picolyl Azide Toolkit; Thermo Fisher Scientific) before analysis by flow cytometry.

### HUDEP-2 cell culture expansion and differentiation

HUDEP-2 cells were provided by Y. Nakamura (RIKEN Institute, Japan). HUDEP-2 cells were expanded in “expansion media” composed of Stemspan SFEM-I (STEMCELL Technologies), hSCF (50 ng/ml; STEMCELL Technologies), EPO (3 U/ml; STEMCELL Technologies), 1 μM dexamethasone (Thermo Fisher Scientific), doxycycline (1 μg/ml; Thermo Fisher Scientific), and 1× penicillin/streptomycin (Thermo Fisher Scientific) at a cell density of 0.1 × 10^6^/ml to 0.8 × 10^6^/ml. Differentiation of HUDEP-2 cells occurred through a two-step culture method. “Phase 1” of differentiation cultured HUDEP-2 cells in IMDM (Thermo Fisher Scientific), 2% fetal bovine serum (Thermo Fisher Scientific), 3% AB human serum (Thermo Fisher Scientific), EPO (3 U/ml), hSCF (50 ng/ml), doxycycline (1 μg/ml), insulin (10 μg/ml; Thermo Fisher Scientific), holo-transferrin (1 mg/ml; Thermo Fisher Scientific), 1× penicillin/streptomycin, and heparin (3 U/ml; Thermo Fisher Scientific) for days 0 to 3 at a cell density of 0.7 × 10^6^/ml to 1.4 × 10^6^/ml. “Phase 2” of differentiation cultured HUDEP-2 cells in the above-specified phase 1 media aside from the removal of hSCF at a cell density of 1 × 10^6^/ml to 2 × 10^6^/ml. At specified times of maturation, 1 × 10^5^ HUDEP-2 cells were spun in a Shandon Cytospin at 400 rpm for 8 min followed by methanol fixation and Wright-Giemsa staining (Thermo Fisher Scientific) for cytospin morphology preparations.

### Retroviral transduction of HUDEP-2 cells

To produce retroviral particles, human embryonic kidney–293 cells were cotransfected with either pMSCV-IRES-GFP (green fluorescent protein) (Addgene #52107) or pMSCV-eIF4E-IRES-GFP (Addgene #18761) in combination with pCL-Ampho (Novusbio) using Polyfect (Qiagen). Supernatants were harvested at 24, 36, and 48 hours after transfection and filtered through a 0.45-μm filter. Pooled viral supernatant was combined with one volume of Retro-X Concentrator (Clontech) per three volumes of supernatant and incubated at 4°C overnight before centrifugation at 1500*g* for 45 min at 4°C. Concentrated viral pellet was resuspended at 1/30th of the original supernatant volume in HUDEP expansion media. HUDEP cells (1 × 10^6^) were resuspended in 2 ml of HUDEP expansion media and combined with 500 μl of concentrated retroviral supernatant and polybrene (8 μg/ml; Thermo Fisher Scientific). The mixture was incubated at room temperature for 20 min before centrifugation at 800*g* for 30 min at 32°C. Virus-containing supernatant was removed, and cells were resuspended in HUDEP expansion media for 48 hours followed by resuspension in fresh media before sorting of transduced cells by GFP expression on a Sony SH800 Cell Sorter. Single-cell clones were obtained per prior described methods (*35*), and eIF4E expression was confirmed by immunoblotting.

### Human primary CD34^+^ expansion and retroviral transduction

Granulocyte colony-stimulating factor–mobilized peripheral blood CD34^+^ mononuclear cells were obtained commercially (STEMCELL Technologies) and expanded as previously described (*60*). Thawed cells were plated on retronectin-coated wells in CD34^+^ expansion media [StemSpan SFEM II, hSCF (100 ng/ml; Peprotech), Flt3 ligand (100 ng/ml; Peprotech), Thrombopoietin (TPO) (50 ng/ml; Peprotech), low-density lipoprotein (10 μg/ml; STEMCELL Technologies), UM171 (35 nM; Benchchem), and penicillin/streptomycin (Thermo Fisher Scientific)] for 48 hours of expansion before retroviral transduction with pMSCV-IRES-GFP or pMSCV-eIF4E-IRES-GFP as described above. Transduced cells were expanded for 6 days before GFP^+^-mediated sorting, followed by an additional 48-hour expansion. Erythroid expansion was then performed as per Hu *et al.* (*28*). CD34^+^ precursors were plated in phase 1 media [IMDM, 2% human peripheral blood plasma (STEMCELL Technologies), 3% human AB serum (STEMCELL Technologies), interleukin-3 (IL-3; 1 ng/ml; STEMCELL Technologies), holo-transferrin (200 μg/ml), heparin (3 IU/ml), insulin (10 μg/ml), hSCF (10 ng/ml), and EPO (3 IU/ml)] for 6 days at 1 × 10^5^/ml. Cells were then replated at 2.5 × 10^5^/ml in phase 2 media (phase 1 with the exception of removal of IL-3) from days 7 to 10, followed by phase 3 media (phase 2 with the exception of removal of hSCF) at 1 × 10^6^/ml for days 12 to 15. Flow cytometry was performed as specified above on a Cytek 4L Aurora followed by analysis on FlowJo Software (BD).

### TMT MS proteomic analysis

Following procedures previously published for quantitative proteomics, immunoblot assays, and analysis of polysome-enriched mRNAs (*61*), we performed the following methods on HUDEP cell maturation. HUDEP cells were expanded and induced to differentiate as described above. Samples of 10 × 10^6^ cells were prepared in triplicate, washed, and snap-frozen at day +3 after differentiation with phase 1 media.

#### Protein extraction and digestion

HUDEP cell pellets were resuspended in a total volume of 50 μl of 50 mM ammonium bicarbonate buffer and 1% Rapigest SF surfactant (Waters) and treated with 25 U of Benzonase (EMD Millipore), with sonication in a water bath for 5 min at 37°C to disaggregate the cells. The suspensions were then incubated for 30 additional minutes at 37°C. After that, samples were centrifugated at 15,000*g* for 10 min. The supernatant was recovered, and protein content in the extracts was determined using a Micro BCA (bicinchoninic acid) Protein Assay Kit (Thermo Fisher Scientific). Aliquots containing 800 μg of protein were treated with 10 mM tris(2-carboxyethyl)phosphine(TCEP) at 56°C for 30 min, followed by a 30-min incubation at room temperature in the dark with 15 mM iodoacetamide. For tryptic digestion, 5% (w/w) L-(tosylamido-2-phenyl) ethyl chloromethyl ketone (TPCK)-trypsin (Thermo Fisher Scientific) was added to the samples. The pH was adjusted to 8.0 with 250 mM ammonium bicarbonate, and the samples were incubated for 12 hours at 37°C. After that, another aliquot of trypsin was added (2%, w/w), and the samples were digested for an additional 6 hours. After this, samples were acidified with formic acid to a final concentration of 5%. The digests were then desalted using a MAX-RP Sep Pak classic C18 cartridge (Waters) following the manufacturer’s protocol. Sep Pak eluates were dried-evaporated in preparation for labeling with TMT reagents.

#### TMT labeling

Dried samples were resuspended in 100 mM tetraethylammonium bicarbonate buffer, and aliquots containing 200 μg of tryptic peptides were labeled according to the TMT 6-plex kit instructions (Thermo Fisher Scientific). All labeled materials were then organized in a six-plex experiment, combining three biological repeats from WT and eIF4E cells, and then desalted using a C18 Sep Pak. The Sep Pak eluates were dried in preparation for fractionation by high-pH reversed-phase chromatography.

#### High-pH reversed-phase chromatography

Labeled samples were fractionated on an AKTA purifier system using a Phenomenex Gemini 5μ C18 110-Å 150 mm by 4.60 mm column, operating at a flow rate of 0.550 ml/min. Buffer A consisted of 20 mM ammonium formate (pH 10), and buffer B consisted of 20 mM ammonium formate in 90% acetonitrile (pH 10). Gradient details were as follows: 1 to 9% B in 3.6 min, 9 to 49% B in 36.3 min, 49 to 70% B in 2.7 min, and 70% B back down to 1% B in 1.8 min. Fifty peptide-containing fractions were collected, evaporated, and resuspended in 0.1% formic acid.

#### MS analysis

Aliquots (containing around 5 μg of digested material) of 10 nonconsecutive chromatographic fractions were run onto a 2-μm, 75 μm × 50 cm PepMap RSLC C18 EasySpray column (Thermo Fisher Scientific) using 3-hour acetonitrile gradients (2 to 25% in 0.1% formic acid) to elute peptides, at a flow rate of 200 nl/min, for analysis in a QExactive Plus Orbitrap MS (Thermo Fisher Scientific) in positive ion mode. MS spectra were acquired between 350 and 1500 mass/charge ratio (*m*/*z*) with a resolution of 70,000. For each MS spectrum, the 10 most intense multiply charged ions over the selected threshold (1.7 × 10^4^) were selected for tandem MS (MS/MS) with an isolation window of 1 *m*/*z*. Precursor ions were fragmented by HCD using stepped relative collision energies of 25, 35, and 40 to ensure efficient generation of sequence ions and TMT reporter ions. MS/MS spectra were acquired in centroid mode with a resolution of 70,000 from *m*/*z* = 100. A dynamic exclusion window was applied that prevented the same *m*/*z* from being selected for 10 s after its acquisition.

#### Peptide and protein identification and TMT quantitation

Peak lists were generated using PAVA in-house software (*62*). All generated peak lists were searched against the human subset of the SwissProt database (SwissProt.2017.11.01), using Protein Prospector (*63*) with the following parameters: Enzyme specificity was set as trypsin, and up to two missed cleavages per peptide were allowed. Carbamidomethylation of cysteine residues and TMT labeling of lysine residues and N terminus of the protein were allowed as fixed modifications. N-acetylation of the N terminus of the protein, loss of protein N-terminal methionine, pyroglutamate formation from peptide N-terminal glutamines, and oxidation of methionine were allowed as variable modifications. Mass tolerance was 20 parts per million (ppm) in MS and 30 ppm in MS/MS. The false-positive rate was estimated by searching the data using a concatenated database that contains the original SwissProt database, as well as a version of each original entry where the sequence has been randomized. A 1% false discovery rate (FDR) was permitted at the protein and peptide level. For quantitation, only unique peptides were considered; peptides common to several proteins were not used for quantitative analysis. Relative quantitation of peptide abundance was performed via calculation of the intensity of reporter ions corresponding to the different TMT labels, present in MS/MS spectra. Intensities were determined by Protein Prospector. Median intensities on each TMT channel for all identified spectra were used to normalize individual intensity values. Relative abundances were calculated as ratios versus the average intensity levels in the three channels corresponding to WT samples. Spectra representing replicate measurements of the same peptide were kept and used to calculate median values of the log_2_ ratios for that peptide. For total protein relative levels, peptide ratios were aggregated to the protein levels using median values of the log_2_ ratios. Cutoff for significant change in protein abundance was set at log_2_(FC) > 0.3.

### Functional annotation of gene set enrichment

Genes significantly increased with increased eIF4E (143 proteins) and in WT (43 proteins) were analyzed using the Database for Annotation, Visualization and Integrated Discovery (DAVID) bioinformatics resource separately. Functional annotation clustering was performed using DAVID defined settings incorporating gene sets from GO (biological process and molecular function) and Uniprot (UP) keywords. The top gene categories statistically significant (FDR < 0.05) were acquired from a combined selected annotation view and subsequently graphed.

### Immunoblot analysis

Immunoblot analysis was performed on samples lysed in radioimmunoprecipitation assay lysis buffer [50 mM tris-HCl (pH 8.0), 150 mM NaCl, 1% NP-40, 0.1% SDS, 0.5% sodium deoxycholate, 1 mM EDTA, and 1 mM dithiothreitol (DTT)] with the addition of phosphatase inhibitory cocktail (Halt Phosphatase Inhibitor Cocktail, Thermo Fisher Scientific) and cOmplete mini proteasome inhibitors (Pierce) using standard procedures. Briefly, lysates were iced and vortexed for 30 min and then centrifuged, and an aliquot of supernatant was used for Bradford assay (or BCA assay) to determine protein concentration. Immunoblot analysis was performed using commercial antibodies listed in table S2. Secondary antibodies used were horseradish peroxidase (HRP) conjugate anti-mouse immunoglobulin G (IgG) (Promega) and HRP conjugate anti-rabbit IgG (Promega). All immunoblots were repeated a minimum of three times with biologically independent samples. Immunoblots were imaged using Chemi-Doc MP (Bio-Rad) or Odyssey (Li-Cor) imaging systems. Quantitation was performed by densitometry using ImageLite Software (Li-Cor).

### Quantitative PCR

RNA was isolated using TRIzol Reagent (Invitrogen) according to the manufacturer’s protocol, followed by quantification on NanoDrop. Samples were processed using a High-capacity Complementary DNA Reverse Transcription kit (Applied Biosystems). Complementary DNA (cDNA) samples were diluted 1:20, and 1 μl of template was used in SYBR green (Thermo Fisher Scientific) for detection of qPCR assay. Gene-specific transcript abundance was normalized to β-actin per sample, and statistical comparison was made on a minimum of triplicate independent experiments. Primer sequences used for analysis are listed in table S3.

### Cell polysome fractionation

HUDEP2 cells at day +3 after differentiation in phase 1 media were washed with cold phosphate-buffered saline and cycloheximide (100 μg/ml; Sigma-Aldrich) for 2 min and then lysed directly in polysome lysis buffer [10 mM tris-HCl (pH 8.0), 140 mM NaCl, 1.5 mM MgCl_2_, 0.25% NP-40, 0.1% Triton X-100, 50 mM DTT, cycloheximide (100 μg/ml), and RNasin] for 20 min. Lysates were spun down for 10 min at 9300*g*, and supernatants were loaded onto a 15 to 45% sucrose gradient. Samples were spun at 37,000 rpm for 2.5 hours at 4°C in a Beckman L8-70M ultracentrifuge. Samples were then separated on a gradient fractionation system (BioComp) to evaluate polysome profiles and collect fractions.

### Analysis of polysomal-associated mRNAs

RNA was isolated from individual fractions using TRIzol LS Reagent (Invitrogen) and spiked with FLuc RNA as an internal control (approximately 0.5 μg in total diluted in TRIzol LS before addition to 28 fractions). RNA quality was checked by nanodrop. A fixed volume of RNA from each fraction was reverse-transcribed to cDNA using a cDNA Reverse Transcription kit (Applied Biosystems). cDNA samples were diluted 1:20, and 1 μl of template was used for qPCR assay. RNA levels were quantified, normalized to internal FLuc as a control, summed across all fractions, analyzed, and presented as percentages of this total.

### CRISPR-mediated knockdown of eIF4E targets

crRNAs were designed using the Broad Institute GPP sgRNA Designer tool (https://portals.broadinstitute.org/gpp/public/analysis-tools/sgrna-design) and gene-specific crRNA synthesized (IDT). crRNA sequences can be found in table S3. Duplex of crRNA:tracrRNA of either gene-specific sequences or human negative control (Alt-R CRISPR-Cas9 Negative Control crRNA #1, IDT) was annealed at 95°C for 5 min followed by cooling at room temperature for 60 min. RNP complex of Nucleofection Enhancer (IDT), crRNA:tracrRNA duplex (120 pmol), and Alt-R S.p. Cas9 Nuclease V3 (104 pmol, IDT) were combined and incubated at room temperature for 10 min before addition of 2.5 μl of Nucleofection solution (Amaxa P3 Nucleofector Kit, Lonza). HUDEP-WT or HUDEP-4E cells at 3.0 × 10^5^ per reaction were nucleofected with the above RNP complex with Nucleofection solution using program “CA-137” and recovered in 100 liters of HUDEP expansion media for 10 min at 37°C before flushing nucleofection cuvette and transferring to culture. HUDEP cells expanded for 48 hours before single-cell colony isolation as listed above and analyzed for gene-specific knockdown using immunoblotting.

### Analysis of 5′UTR characteristics

Data on analysis of 5′UTR characteristics can be found in table S4. Characteristics were derived using RNAfold in ViennaRNA package (version 2.4.12, default parameters). In silico prediction of the alignment of the identified motif in comparison to verified recognition motifs of RNA binding proteins was performed using RBPsuite (www.csbio.sjtu.edu.cn/bioinf/RBPsuite/) (*43*).

### Identification of conserved 5′UTR motif

For the motif discovery, the genes up-regulated in the eif4e samples and WT samples were extracted. For each gene, the expression of their isoforms was quantified using salmon and tximport in R. The isoform with the highest abundance was chosen for each gene. For the corresponding gene sets, their 5′UTR sequences were extracted from a UCSC hg38 genome. Both sets of sequences were cleaned using the “seqinr” package in R.

For both sets of sequences, a set of negative control was made by shuffling the sequencing using the python package “ushuffle” (*64*), which allows us to preserve the doublet size of the sequences. All four sets of sequences were analyzed using the perl package FIRE (*40*). FIRE uses mutual information to test for the conservation of motifs across sequences when compared to their negative controls. FIRE also quantifies a conservation index if a motif is found and evaluates the orientation bias of the motif. The presence of a strand bias helps us distinguish an RNA motif from a DNA motif.

### 5′UTR luciferase reporter assays

5′UTRs were synthesized (gBlock; IDT) and cloned into the pGL3-SV40 (Fluc) construct vector. Sequences of 5′UTR and variants are located in table S3. HUDEP-2 cell lines were nucleofected (Amaxa 4D; Lonza) simultaneously on day +3 after phase 1 differentiation with pGL3-SV40 vectors (3 μg) containing the specified 5′UTR of interest and the pRL (Rluc) (5 ng) plasmid using protocol “ER-100” and kit P3 at 9 × 10^5^ cells per reaction. Cells were collected 16 hours after nucleofection and assayed using the dual luciferase kit (Promega) on the Promega Explorer detection instrument. Correlation of luciferase activity was normalized to Fluc and Rluc RNA levels as quantified by qPCR with reverse transcription, and then firefly luciferase activity was normalized to *Renilla* luciferase activity. Primer sequences for amplification of Fluc and Rluc can be found in table S3.

## Notes

View/request a protocol for this paper from *Bio-protocol*.
