# Supplementary material for: Regulation of eIF4E guides a unique translational program to control erythroid maturation
Source: Sci Adv. 2022 Dec 23;8(51):eadd3942. doi: 10.1126/sciadv.add3942 (PMC9788769; doi:10.1126/sciadv.add3942)

Science Advances
AAAS

Supplementary Materials for

Regulation of eIF4E guides a unique translational program to control erythroid maturation

Craig M. Forester *et al.*

Corresponding author: Davide Ruggero, davide.ruggero@ucsf.edu;
Craig M. Forester, craig.forester@cuanschutz.edu

Sci. Adv. **8**, eadd3942 (2022)
DOI: 10.1126/sciadv.add3942

The PDF file includes:

Figs. S1 to S6
Legends for tables S1 to S4

Other Supplementary Material for this manuscript includes the following:

Tables S1 to S4

Supplemental Figures and Legends

Figure S1. Fluctuations in abundance of mTOR effectors in erythropoiesis. A. Data extracted from prior proteomics dataset of erythroid maturation(*20*) plotted log10 fold change comparing protein abundance at specified stages relative to 'Prog1' early progenitor state. **B.** Data extracted from prior transcriptomics dataset of erythroid maturation(*21*) plotted log10 fold change at specified stages relative to BFU-E. **C.** Representative flow plots demonstrating percent phosphorylation versus total protein of phospho-4EBP1 and phospho-rpS6 after 1 hour treatment with PP242 at specified concentrations in HPC-7 cells. **D.** Western blot analysis of phosphorylation of 4EBP1 and rpS6 1hr post treatment with 2μM of PP242. Isoforms (α,β and γ) depict mobility forms of 4EBP1 dependent upon posttranslational phosphorylation. **E.** Representative flow plots demonstrating percent phosphorylation versus total protein of phospho-4EBP1 and phospho-rpS6 at delineated stages of erythroid maturation used to assess data aggregated in Figure 1C and 1E.

Figure S2. Establishing HUDEP model to study effect of eIF4E expression on erythroid maturation. A. Percent phosphorylation depicted as demonstrated in Figure S1 with incorporation of phosphorylated upstream $mTOR^{Ser2448}$. **B-C.** Comparison of phospho-antibody (B) and total protein (C) signal by MFI of 4EBP1 and rpS6. * Indicates comparison of 4EBP1 signal in Precursor Stage 1 compared to remaining stages ($p<0.05$). # Indicates comparison of rpS6 signal in Precursor Stage 1 compared to remaining stages ($p<0.05$). **D.** Western blot analysis of eIF4E abundance and 4EBP1 abundance/phosphorylation at days post induction of erythroid maturation in HUDEP-WT cells. **E.** Flow cytometry strategy for isolating markers of early erythroid precursors in GFP+ transduced HUDEP-2 cells. GFP+ cells were subdivided by CD71+, FSC into C ($CD71^{lo}$, FSC^{lo}), B ($CD71^{hi}$, FSC^{lo}) and A ($CD71^{hi}$, FSC^{hi})(*27*) followed by gating into markers of early erythroid precursor phases of CD71A, CD34+, CD105+, CD71A, CD34-, CD105+ and CD71A, CD34+, CD105+, cKit+(*26*). **F.** Representative flow cytometry plots comparing HUDEP-WT and HUDEP-eIF4E cells in expansion media (Day 0). **G-I.** Comparison of HUDEP-WT and HUDEP-eIF4E cells in precursor expansion media by G. CD71, FSC, H. CD34, CD105 and I. cKit gating according to strategy in Fig S2E $p<0.05$, N=4.

Figure S3. Increased eIF4E impairs HUDEP and Human CD34+ primary erythroid maturation and imparts a specific translational program. A. Quantitation of eIF4E overexpression in retrovirally transduced and GFP+ sorted primary human CD34+ MNCs derived from GCSF mobilization. "WT" denotes transduction with pMSCV IRES GFP while "eIF4E" denotes transduction with pMSCV eIF4E IRES GFP. *$p<0.05$ by unpaired Student's t-test (N=4). **B**. Representative flow cytometry plots at specified time point depicting GFP+, CD235a+ cell populations further gated by staining for CD49d (α4-integrin) vs CD233 (Band3). **C**. Analysis of gated populations at respective timepoints. *$p<0.05$, unpaired Student's t-test. N=3. **D.** Quantitation of flow cytometry defined differences in maturation between WT and eIF4E HUDEP-2 cells at Day+3 by CD71, FSC; CD34, CD105 and cKit. * $p <0.05$, N=3**. E.** GOTerm class enrichment of genes upregulated (log2 FC<-0.3) in HUDEP-WT vs eIF4E (upper) and GOTerm class enrichment of genes upregulated (log2 FC>0.3) in HUDEP-4E vs WT (lower). **F.** Data extracted from prior transcriptomics dataset of erythroid maturation(*21*) plotted log10 fold change at specified stages relative to BFU-E. **G.** Data extracted from prior proteomics dataset of erythroid maturation(*20*) plotted log10 fold change comparing protein abundance at specified stages relative to 'Prog1' early progenitor state.

Figure S4. Minimal rescue of PTPN6 or Igf2bp1 knockdown on reduction of CD105 or cKit cell surface markers. **A.** QPCR analysis of PTPN6, Igf2bp1, eIF4E and 4EBP1 across maturation Day 0-3. Displayed as actin-normalized fold change in comparison to respective Day

0 values per mRNA. * indicates $p<0.05$ in comparison to Day 0 transcript abundance by unpaired Student's t-test. **B**. Western blot showing fluctuations in protein abundance across Day 0-3 in wild-type HUDEP maturation. **C.** RT-QPCR of transcript abundance of eIF4E-upregulated genes expressed as fold change relative to HUDEP-WT at Day +3 post Maturation induction. $p < 0.05$ by Student's t-test. **D.** Analysis of protein stability by western blot at 0,2,4,6 and 8 hours post treatment with cycloheximide (100μg/ml) of PTPN6, Igf2bp1, eIF4E and Actin. **E.** Quantitation of actin-normalized protein abundance relative to t=0 hours per respective protein and HUDEP background. * $q<0.05$ by unpaired t test, with a Desired FDR (Q) of 1.00%. **F.** Representative western blot showing expression of eIF4E, PTPN6, Igf2bp1, total and phospho-4EBP1 at Day 0 in HUDEP-WT vs HUDEP-eIF4E. eIF4E expression quantified by densitometry in HUDEP-eIF4E vs WT (log2FC=2.68, SD=0.08345) in GFP+, retrovirally transduced HUDEP cells. N=4. G. Fluorescence MFI Click-conjugated Alexa555 fluorophore as a measure of global protein synthesis activity 2hrs after addition of OP-Puromycin (30 μM) to HUDEP-WT or HUDEP-4E at Day +3 post maturation induction with Phase 1 media. **H.** QPCR of *Actin* at specified polysome fractions in HUDEP WT vs eIF4E. * $q<0.05$ unpaired t test, employing a False Discovery Rate (FDR) with a Two-stage step-up (Benjamini, Krieger and Yekutieli) with a Desired FDR (Q) of 1.00%.

Figure S5. Analysis of CRISPR target efficacy and erythroid gene rescue. A. Western blot depicting PTPN6 and Igf2bp1 expression in single cell clones of CRISPR gene edited HUDEP-eIF4E cells derived from two unique targeting gRNA (#1 and #2) per gene. **B**. QPCR of array of mRNA associated with mature erythroid differentiation including β-globin, Spectrin A, Ankyrin and Band 4.2 at Day 4. Actin-normalized mRNA transcript in HUDEP-eIF4E with noncoding CRISPR guide (HUDEP-4E Neg) and HUDEP-eIF4E edited clones against PTPN6 and Igf2bp1 were compared to abundance in HUDEP-WT with noncoding CRISPR guide (HUDEP-WT Neg). *$p<0.05$ by Student's t-test (N=5).

Figure S6. Analysis of conserved motif in 5' UTR of WT upregulated genes. A. GC content (% sequence) of 5' UTR sequences in WT and eIF4E-upregulated genes (Median WT 63.98% vs eIF4E 66.23%). * $p = 0.0077$ **B.** Free Energy determined by MFE (kcal/mol) of 5' UTR sequences in WT and eIF4E-upregulated genes (Median WT -110.9 vs eIF4E -84.4). *$p = 0.0288$. **C.** Sequence length (nucleotide number) of 5' UTR sequences in WT and eIF4E-upregulated genes (Median WT 310 vs eIF4E 203). * $p = 0.0146$. Statistical significance determined by Komogorov-Smirnov test. Data on 5' UTR derived from RNAfold in ViennaRNA package (version 2.4.12, default parameters). **D.** Conserved motif identified with FIRE in 5'UTR of eIF4E-upregulated genes did not demonstrate enrichment in 5'UTR of WT-upregulated genes. **E.** Graphical representation of in silico 5'UTR structure prediction based upon minimum free energy of -148.20 kcal/mol of Igf2bp1 performed on RNAfold WebServer. Red box indicates position of identified conserved motive. **F.** Graphical representation of in silico 5'UTR structure prediction based upon minimum free energy of -98.3kcal/mol of Igf2bp1performed on RNAfold WebServer. Red box indicates position of identified conserved motive.

Supplemental Table Legend

Table 1: Itemized list of TMT mass spectrometry analysis of identified proteins corresponding to data represented in Figure 3B. Individual values of replicates (n=3) for each sample in either HUDEP-WT (wt) or HUDEP-4E (eIF4E) genetic backgrounds including unique isolate peptides, percent coverage, intensity quantitation and estimation of average log2 fold change between HUDEP-eIF4E and HUDEP-WT conditions.

Table 2: Itemized list of antibodies used in both immunoblotting and flow-cytometry based experiments.

Table 3: Itemized list of oligonucleotide sequences employed including sequences used for quantitative mRNA analysis of candidate genes and luciferase reporter abundance, crRNA sequences employed in gene editing experiments for targeted knockdown in HUDEP cells and sequences of candidate gene 5' UTR cloned into pGL-SV40 and respective C-rich motif nucleotide transversions (tv) and deletions (del).

Table 4: Itemized list of 5' UTR sequences of genes upregulated in HUDEP-WT or HUDEP-4E cells 3 days post induction of maturation in Phase 1 media. Sheets listed itemize respective minimum free energy (mfe), GC content (GC), nucleotide length (length).

Supplemental Figure 1.

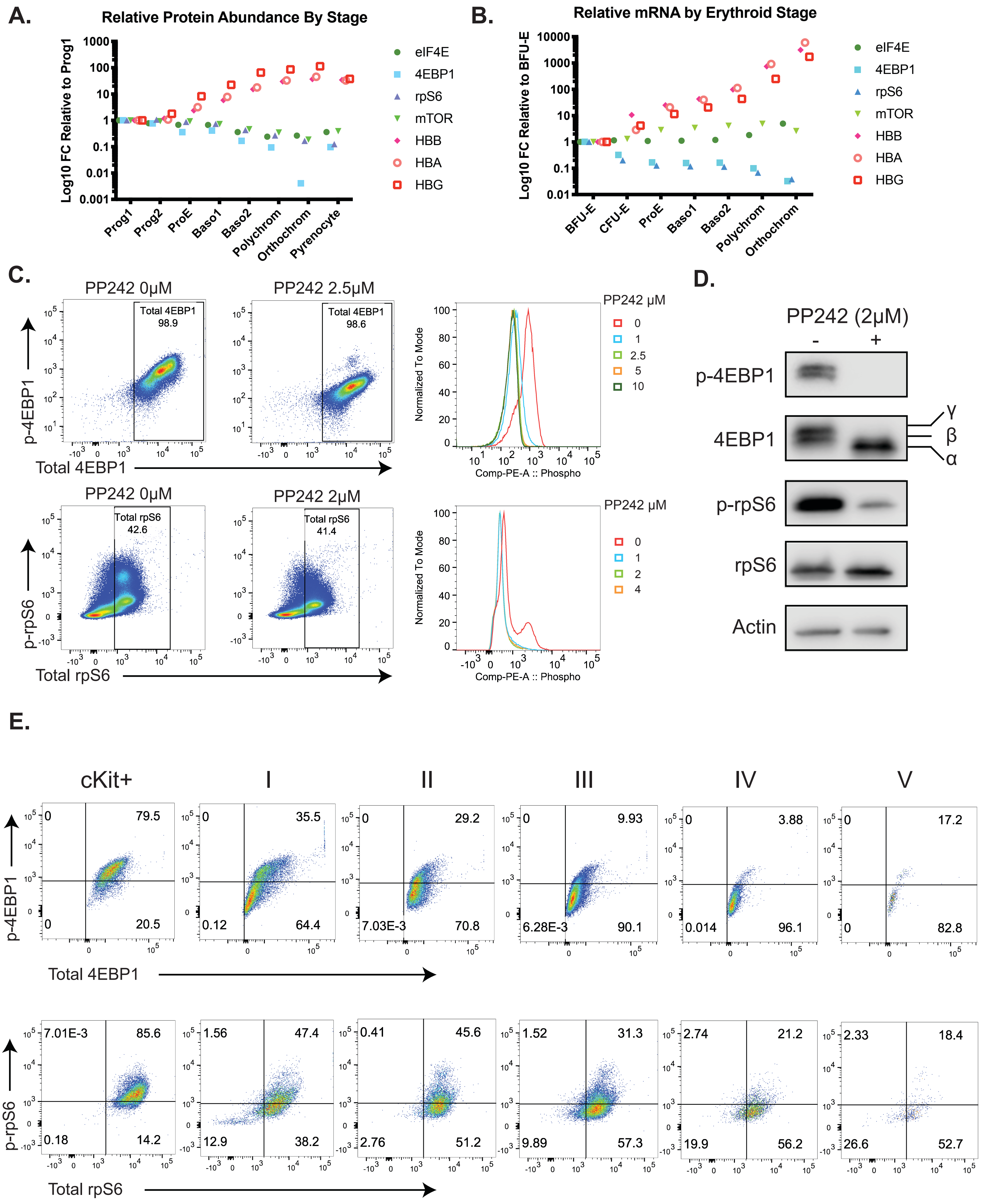

Supplemental Figure 2.

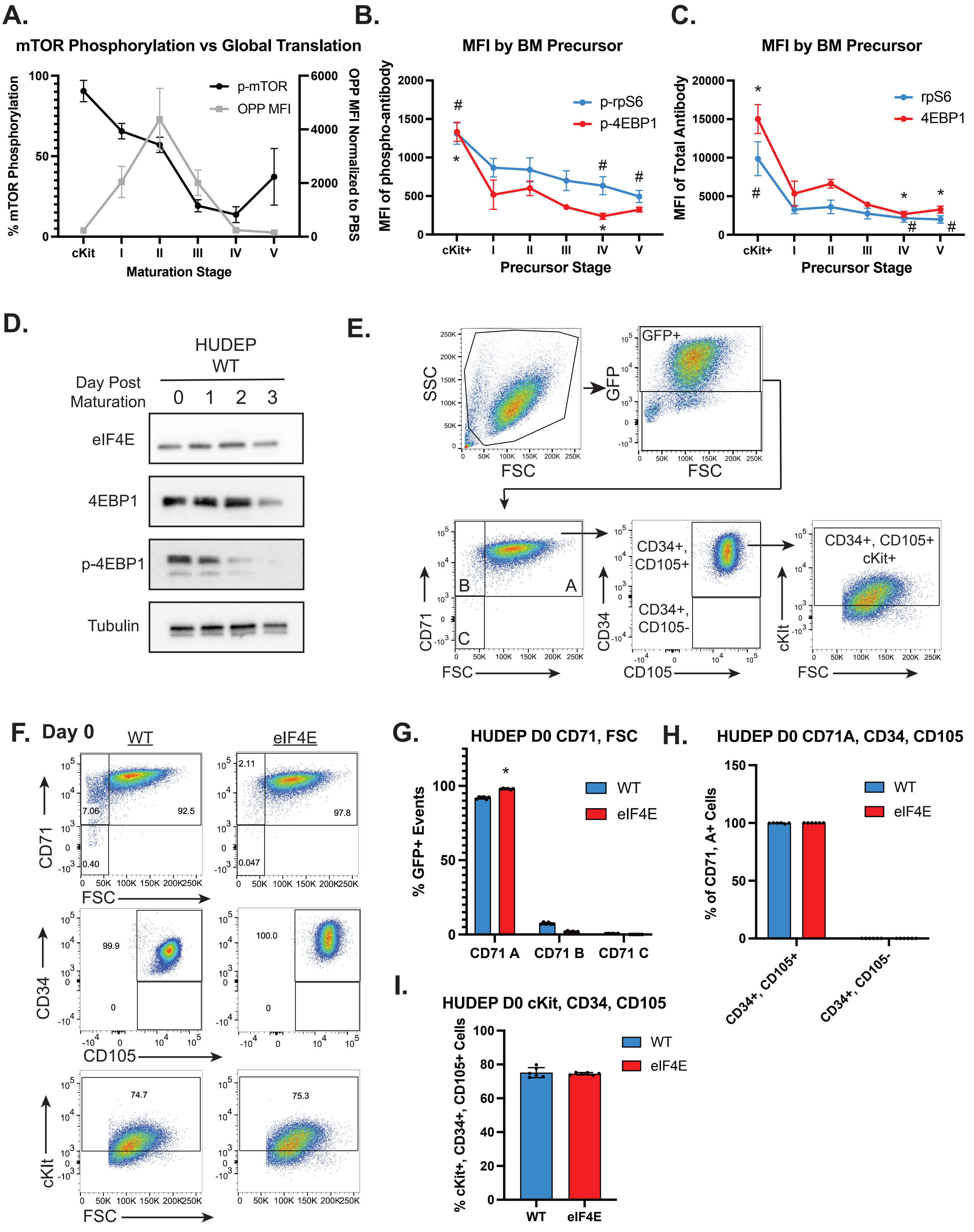

Supplemental Figure 3.

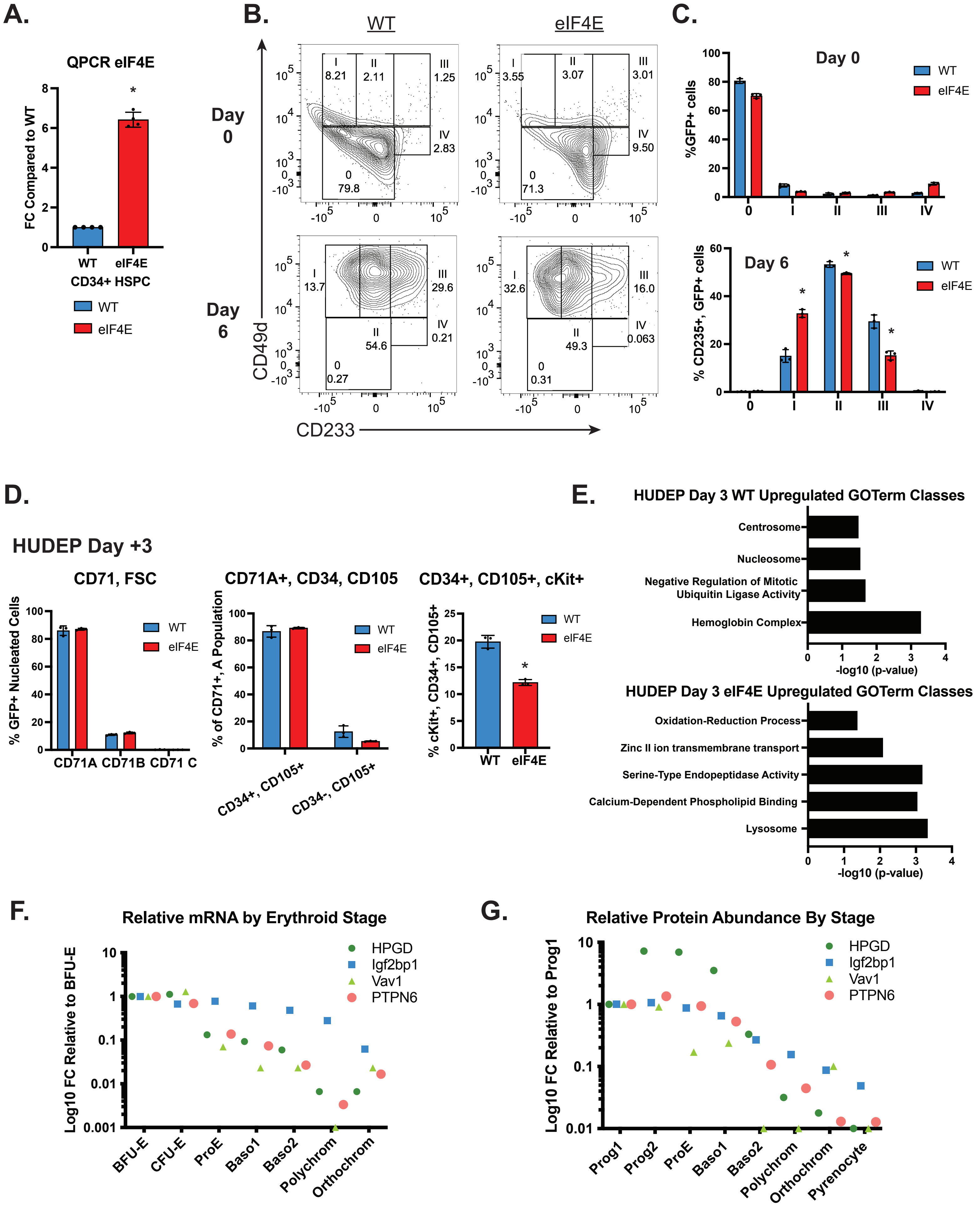

Supplemental Figure 4.

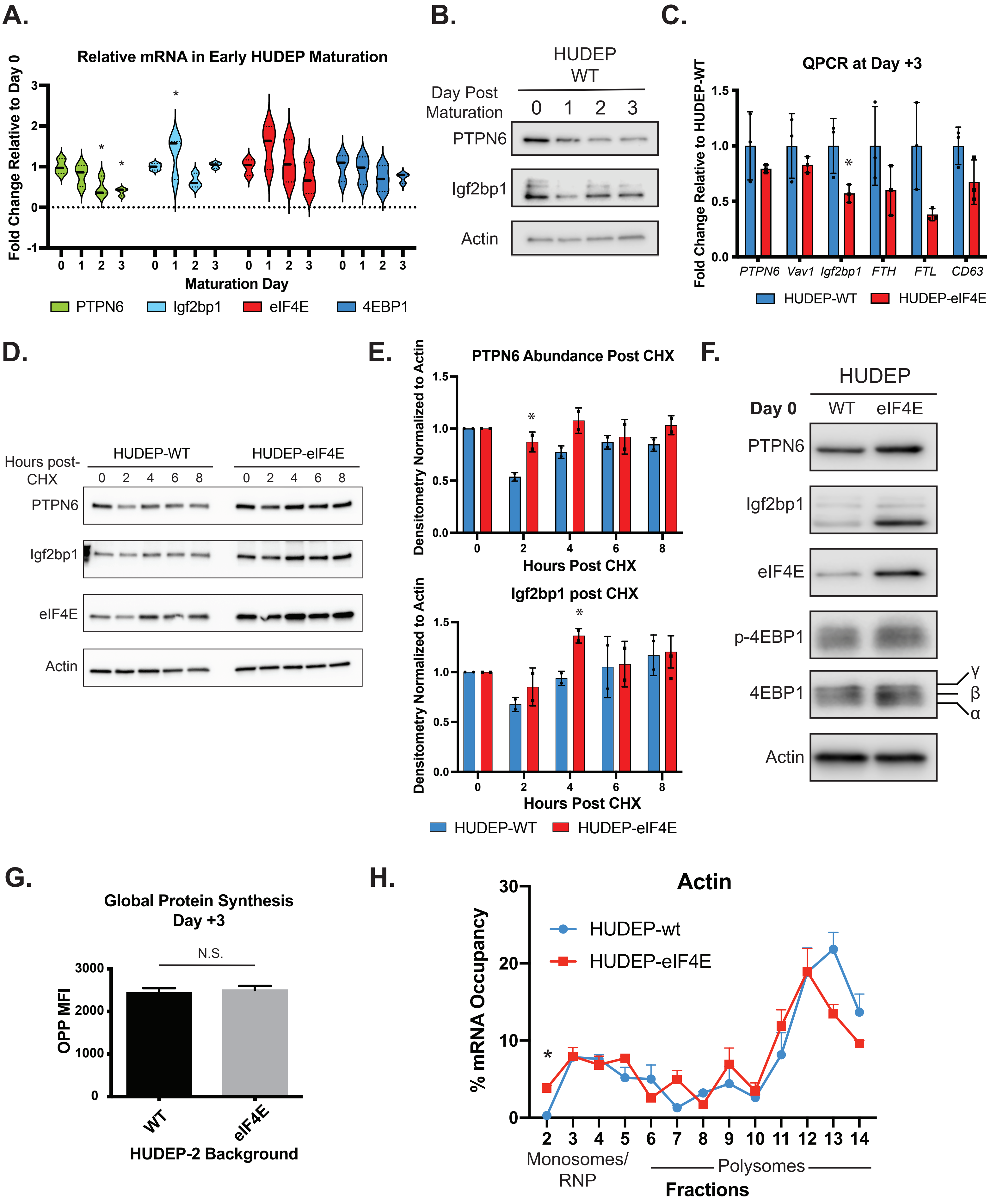

Supplemental Figure 5.

A.

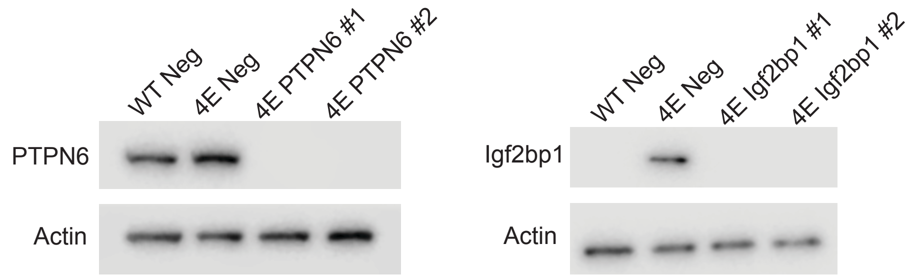

B.

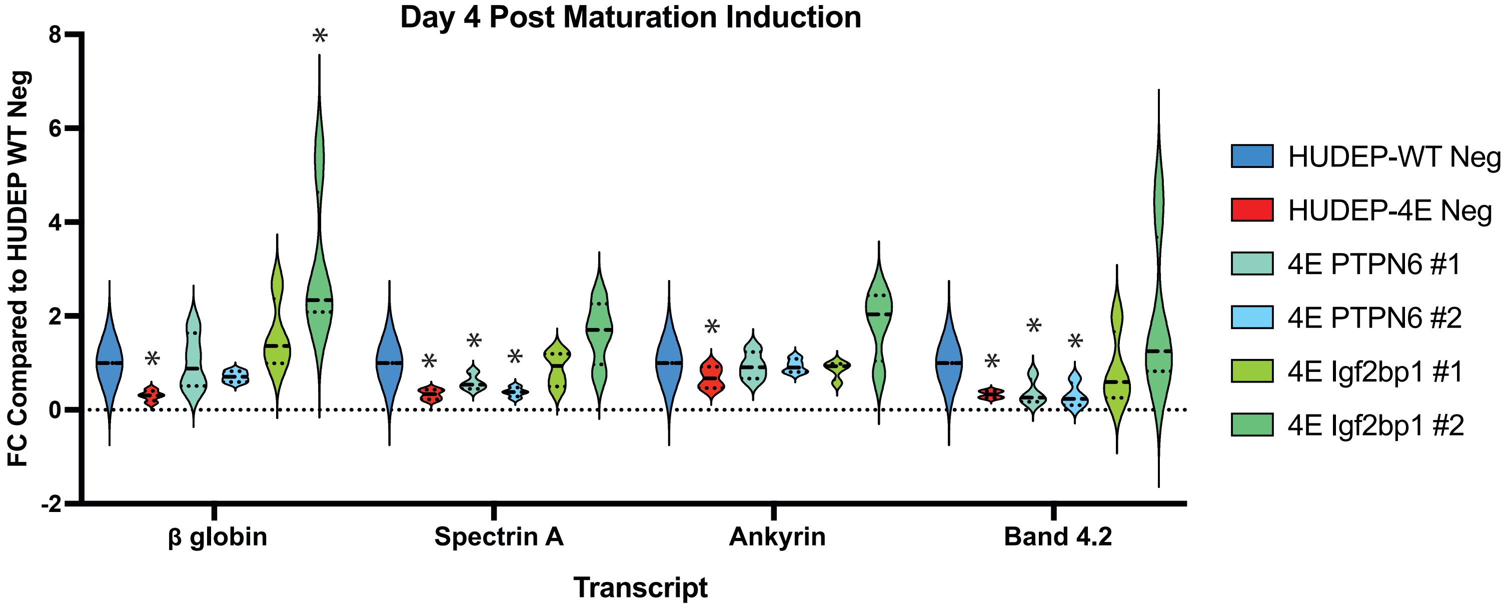

Supplemental Figure 6.

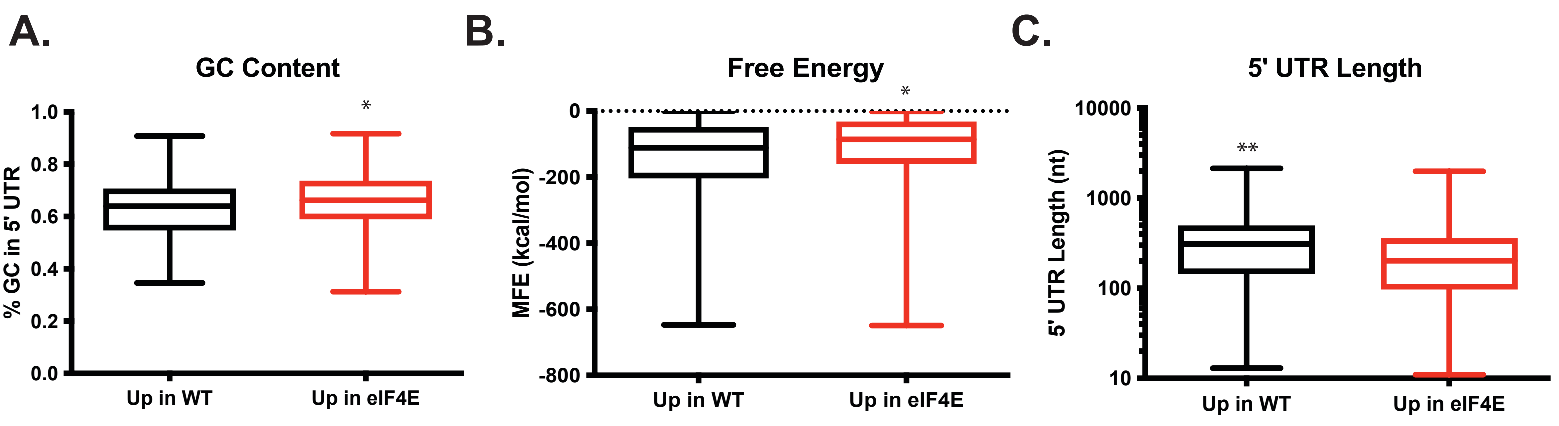

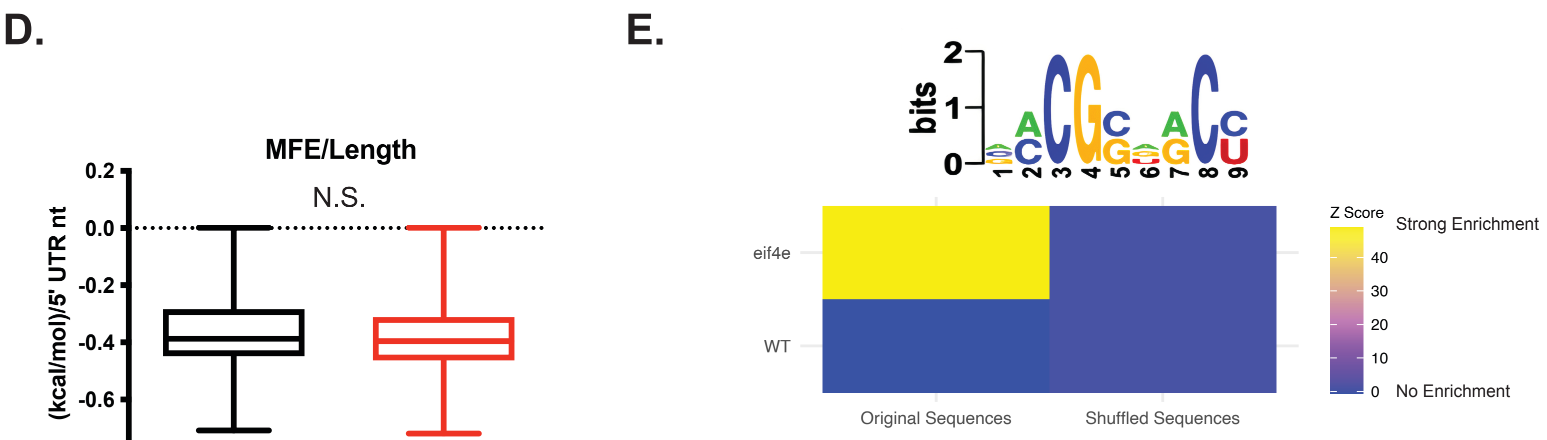

5'UTR Upregulated in	z-score	p-value	robustness	conservation index
eIF4E	48.9	2.72e-520	10/10	0.94
WT	-0.523	0.3479477	0/10	N/A

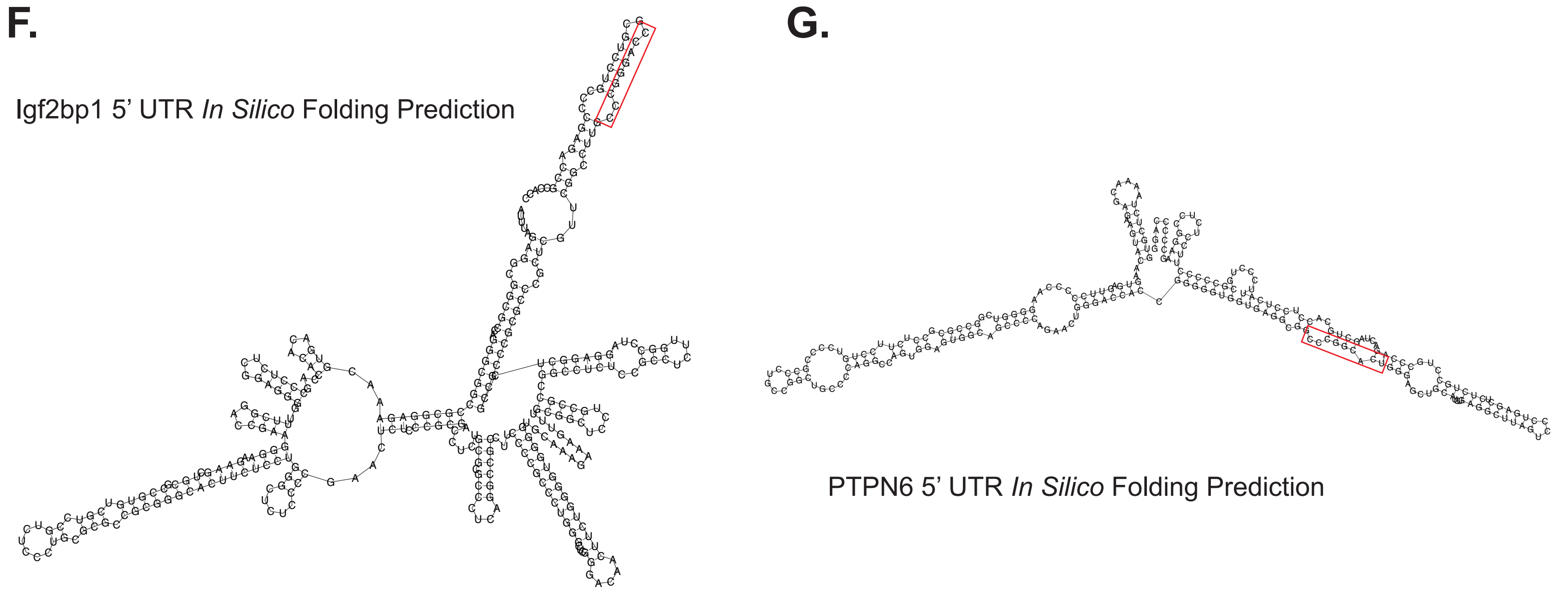

Supplement: Supplementary file 1 — Figs. S1 to S6 [file sciadv.add3942_sm.pdf]
